# The role of mindfulness training supported by virtual reality in the nonpharmacological treatment of schizophrenia-research design

**DOI:** 10.1186/s12991-025-00587-5

**Published:** 2025-08-13

**Authors:** Andrzej Cechnicki, Adrian Chrobak, Iga Plencler, Przemysław Stankiewicz, Aneta Kalisz, Piotr Błądziński, Dawid Kruk, Stanisław Radoń, Bernadetta Szewczyk, Agata Faron-Górecka, Michał Korostyński, Marcin Siwek

**Affiliations:** 1 Chair of Psychiatry, Community Psychiatry and Psychosis Research Center, Jagiellonian University Medical College, Cracow, Poland; 2 Department of Adult Psychiatry, Jagiellonian University Medical College, Cracow, Poland; 3 Doctoral School of Medical and Health Sciences, Jagiellonian University Medical College, Cracow, Poland; 4 Faculty of Social Sciences, The Pontifical University of John Paul II, Cracow, Poland; 5 Department of Neurobiology, Polish Academy of Sciences, Maj Institute of Pharmacology, Cracow, Poland; 6 Department of Pharmacology, Polish Academy of Sciences, Maj Institute of Pharmacology, Cracow, Poland; 7 Department of Molecular Neuropharmacology, Polish Academy of Sciences, Maj Institute of Pharmacology, Cracow, Poland; 8 Chair of Psychiatry, Department of Affective Disorders, Jagiellonian University Medical College, Cracow, Poland

**Keywords:** Mindfulness, Virtual reality, Schizophrenia

## Abstract

This paper presents the outcome of a clinical trial planning process developed during multicenter, multidisciplinary seminars with prospective collaborators. The study protocol, designed according to the Oxford Quality Assessment System, outlines a randomized controlled trial (RCT) evaluating a novel Virtual Reality-based Mindfulness Skills Training (VR-MST) versus standard, non-VR MST in patients with schizophrenia (SZ). The trial aims to assess the effects of VR-MST on clinical outcomes, stress-related biomarkers, and gene expression.

Eligibility criteria include: (1) SZ diagnosis based on ICD-10 and DSM-5 (2), psychotic symptom severity below 75 on the PANSS (moderately ill), and (3) age between 25 and 50 years. The protocol defines procedures for participant withdrawal and managing adverse events. The design tests both specific and general hypotheses, with pre- and post-intervention assessments in both groups, and additional pre-/post-session measurements in the VR group. Assessments span biological, symptomatic, and cognitive domains, using both objective and subjective measures.

This study may inform clinical practice by introducing a novel, engaging, evidence-based, nonpharmacological intervention. VR-MST could support stress reduction, enhance cognitive functioning, and improve daily life in SZ patients. The design is grounded in prior pilot studies, literature reviews, and clinical expertise, aiming to provide a scalable and impactful therapeutic tool.

## Introduction

An analysis of PubMed records from the last 25 years revealed that nearly 4,000 publications are dedicated exclusively to pharmacotherapy for schizophrenia, comprising clinical trials and randomized controlled trials (RCTs). In contrast, nonpharmacological interventions with similar methodologies account for only approximately 1,300 publications. These findings point to a significant imbalance, with pharmacotherapy research vastly outnumbering nonpharmacological studies. Consequently, there is an urgent need to investigate nonpharmacological interventions for schizophrenia to enhance existing knowledge and develop appropriate standards for supportive treatment. However, conducting studies on nonpharmacological interventions may present various methodological challenges.

The complexity of the interrelationships and challenges in interpreting the results of nonpharmacological interventions for schizophrenia can lead to methodological errors. One way to avoid seemingly correct results is to prepare the research design carefully. To avoid false positive outcomes, a well-planned research design is recommended. The authors present the outcome of a year-long research seminar in a broad multicenter and multispecialty group of prospective investigators and intervention developers on the preparation of a research project.

Recent studies highlight the increasing relevance of digital and interactive technologies in the field of mental health, especially in supporting health literacy and engagement among younger populations. Mancone [1] provide a comprehensive review of how digital tools can enhance adolescent health outcomes by fostering interactivity, motivation, and individualized learning processes. These findings support the integration of virtual reality (VR) and other immersive technologies into mental health interventions, aligning with the goals of the present study to explore innovative, nonpharmacological approaches in schizophrenia treatment.

Schizophrenia is a multidimensional disease with a complex etiopathogenesis and diverse courses [2]. The vulnerability‒stress‒inflammation model of this illness [3], which involves biological and environmental factors, is believed to best reflect the current state of knowledge. Exposure to stress leads to an increased inflammatory response (release of proinflammatory cytokines), oxidative stress and decreased levels of neurotrophic factors, i.e., BDNF. The group of these parameters whose levels change in SZ patients in response to stressors will be referred to as “stress-related markers” in our project.

Markers of inflammation and oxidative stress constitute an important group of biological parameters that we plan to assess. Chronic inflammation and increased oxidative stress are present in patients with schizophrenia in the premorbid period before the onset of treatment, and their levels are increased during periods of exacerbation of the illness in the course of a psychotic episode. Moreover, a tendency toward increased inflammation has been observed with illness progression [4]. The CNS is particularly vulnerable to both of these processes because of its high oxygen demand, increased production of free oxygen species, reduced antioxidant response, and lipid-rich structure susceptible to oxidation [5]. Elevated levels of oxidative stress indicators, particularly lipid peroxidation markers such as thiobarbituric acid reactive substances (TBARS), constitute one of the most replicable biomarkers in SZ patients [6]. Studies highlight the important role of inflammation in the pathophysiology of schizophrenia, indicating elevated C-reactive protein (CRP) and cytokine levels (e.g., TNF-alpha, IL-6, and IL-8) in this disease [6–8]. One of the biological markers we will measure is brain-derived neurotrophic factor (BDNF), the role of which in schizophrenia is still unclear. Its plasma concentration in people with schizophrenia is lower than that in healthy subjects [9]. Its concentration was found to correlate positively with some aspects of cognitive functioning [10]. A correlation between BDNF concentration and the severity of positive [11] or negative [12] symptoms was also shown several times, but it was not found in one meta-analysis [13]. Other studies suggest that there may be a negative correlation between BDNF concentration and the severity of depressive symptoms in patients with schizophrenia [14]. Furthermore, studies have shown a negative correlation between CRP and BDNF and a potential association between this protein and the cognitive functioning of SZ patients [15].

Owing to the frequent phenomenon of drug resistance and insufficient remission in patients with schizophrenia, an increasing number of studies are looking for new nonpharmacological methods for the treatment of this disorder [16]. As current research indicates, among the effective methods for managing the level of perceived stress are techniques based on the practice of mindfulness. The results of previous studies on MBSR in healthy individuals are relatively consistent. They have a strong effect on stress reduction and moderate effects on the reduction of anxiety, distress, depression and quality of life [17]. A review paper on mindfulness-based interventions (MBIs) for individuals at risk of psychosis and those in the first episode of psychosis described a reduction in anxiety and sadness, improved quality of life and, in some cases, a reduction in psychotic symptoms [18].

In the most recent meta-analysis of MBIs in schizophrenia, an improvement with a large effect size was observed in general functioning, with a moderate effect size in general psychopathology and a small effect size in terms of positive and negative symptoms [19]. The biological mechanism of mindfulness practices has not been clearly defined thus far. It has been suggested that it has an impact on the reduction of stress and on the activity of the hypothalamic‒pituitary‒adrenal axis and both the autonomic nervous system and immune system [20, 21], which is associated with an increase in the level of BDNF. Black [22] indicated that an important biological mechanism of mindfulness training is its influence on gene expression.

The use of mindfulness-based techniques leads to a reduction in the level of perceived threat in response to stressful stimuli, resulting in decreased sympathetic nervous system activity [23]. Through beta-adrenergic receptors, this system influences the expression of transcription factors, i.e., NF-kappaB and CREB, which control the expression of numerous groups of genes, including those encoding proteins related to the inflammatory response. The vulnerability-stress-inflammation model (3) indicates a key role of stress in exacerbating inflammatory processes in schizophrenia.

**Fig. 1:**
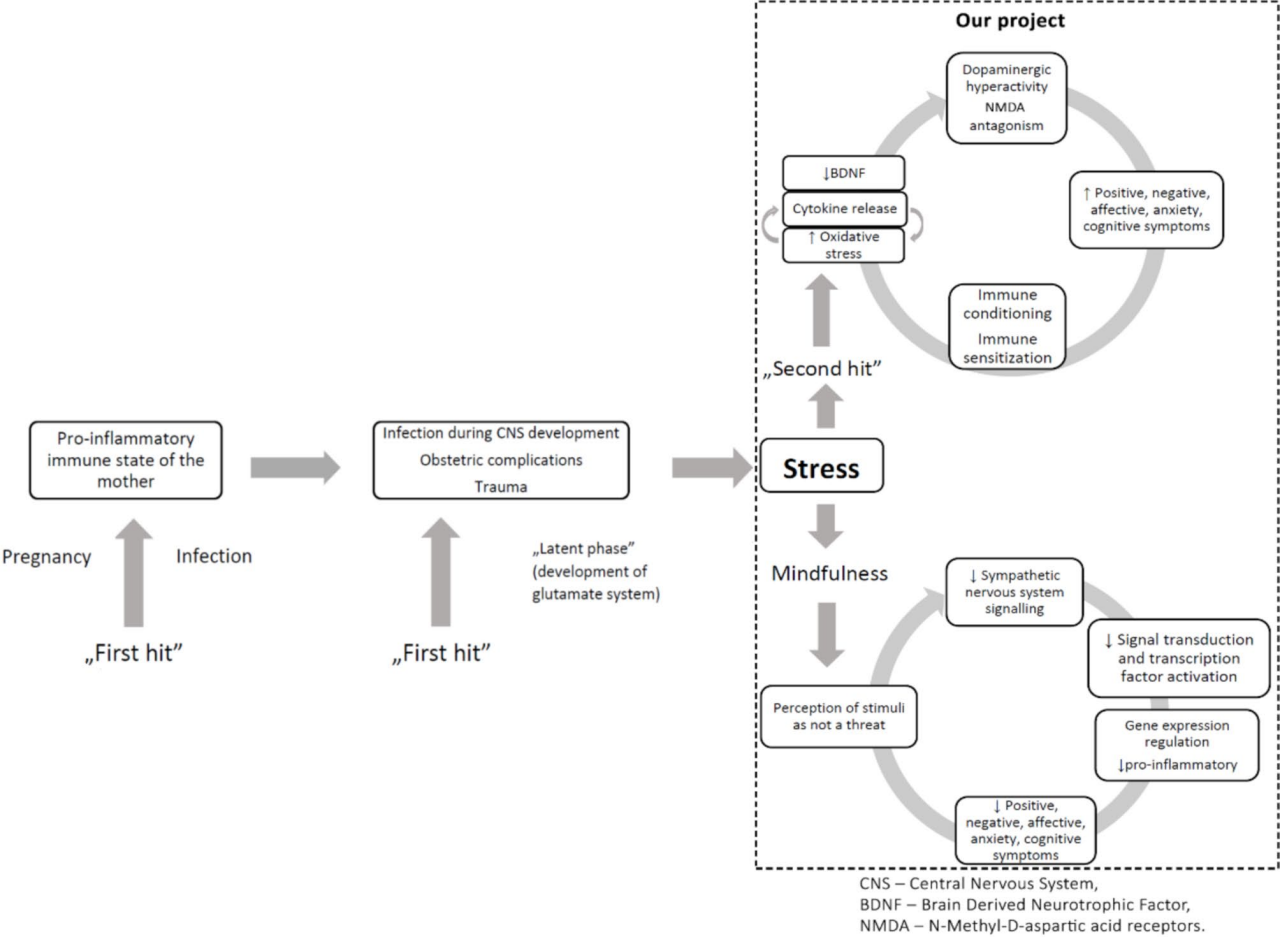
The theory modified Müller’s [3] vulnerability‒stress‒inflammation model incorporating the role of mindfulness training according to Black [22] with the research areas concerning our project

Chadwick [24] suggested that the stress resulting from experiencing psychosis is not a direct result of the content of the symptoms themselves but is related to how the individual relates to the experience. Negative perceptions of the ability to control symptoms, their presence and their content increase stress. At the same time, avoidance of experiencing symptoms increases the negative feelings associated with their presence; in this case, the use of mindfulness techniques also serves as exposure therapy. Owing to the skills acquired during mindfulness training, individuals learn to accept a symptom as a temporary mental event, consequently lowering the level of distress [23]. We hypothesize that one of the gene groups showing abundant changes in patients with a diagnosis of schizophrenia in response to MST is the group of genes associated with the inflammatory response. Studies on genome-wide transcriptional changes will enable the identification of new groups of genes and biological mechanisms associated with their regulation **(**Fig. 1**).**

According to the model, there are two key groups of processes, called ‘hits’, affecting the development of the pathophysiology of schizophrenia and the severity of its symptoms. ‘First hit’ are factors affecting early CNS development, i.e., infections during pregnancy, perinatal complications, and trauma. They lead to an increased susceptibility to stress, which is the so-called ‘second hit’. This project will focus on the “second hit”. The processes that can be classified under this banner lead to disruptions in neurotransmission (imbalances in dopaminergic and glutamatergic transmission), immunological sensitization, a proinflammatory response, increased oxidative stress and decreased levels of neurotrophic factors, i.e., BDNF. They result in increased psychopathological symptoms (i.e., positive, negative and affective symptoms). Mindfulness training leads to a reduction in the level of perceived threat in response to stressful stimuli. This leads to a decrease in sympathetic nervous system activity, which, in turn, causes a reduction in signal transduction and changes in the activity of transcription factors, including those encoding proteins related to the inflammatory response. These changes are associated with decreased severity of the psychopathological symptoms of schizophrenia.

The current literature highlights the great potential of VR techniques used in mindfulness therapy. This method supports such an intervention by enabling a fuller focus on the therapeutic process [25], increasing participants’ involvement [26] and adherence [27]. VR is very engaging and directs attention away from the outside world [25]. VR has been studied in psychotic patients multiple times, including for therapeutic purposes, and is considered a safe method [28, 29].

Previous research has shown that VR-based interventions can effectively reduce symptoms of paranoia, anxiety, and social avoidance in patients with schizophrenia [30, 31]. For example, Pot-Kolder [31] demonstrated that VR-based cognitive behavioral therapy for psychosis (VR-CBTp) led to significant improvements in social functioning and reduced paranoid ideation. Similarly, Freeman [30] reported that a brief VR cognitive therapy session significantly reduced anxious thoughts in social situations. These findings support the relevance of integrating immersive technologies in psychotherapeutic approaches for schizophrenia and provide a strong foundation for our use of VR in mindfulness-based training.

VR in combination with mindfulness training has been used in several recent studies, most of which have been carried out on healthy individuals, but there have also been attempts to target interventions for patients, e.g., those with a generalized anxiety disorder [27]. However, this method has not been used in a group of patients with psychotic symptoms in combination with dedicated VR-MST except in our pilot study, which was the world’s first report on the use of mindfulness and VR as complementary therapeutic methods for patients diagnosed with schizophrenia [32, 33]. The choice of the VR environment for the MST intervention is based on attention restoration theory (ART) [34], which assumes that the natural environment has a regenerative impact on guided attention resources, which has been confirmed in other types of interventions utilizing VR [35]. We hypothesize that in the designed intervention, VR enables schizophrenia patients with attention deficits to engage more fully in mindfulness exercises. Given that the positive impact of the virtual natural environment depends on the preferences of the viewer, we plan to create several scenery-differentiated versions of the training [36].

### Proposed study design

The objective of the project is to measure the impact of our innovative procedure of mindfulness skills training (MST) in virtual reality (VR, VR-MST) on the relationships among stress, psychopathological symptoms, cognitive and social functioning and stress-related markers in a group of patients diagnosed with schizophrenia (SZ). Changes in these factors will be assessed in three randomized groups: classic mindfulness skills training (non-VR-MST), the VR-MST and standard treatment as usual (TAU). The biological measurements in our study will include analyses of stress-related markers (brain-derived neurotrophic factor (BDNF), inflammatory markers, oxidative stress indicators, cortisol and stress-related gene expression evaluation through transcriptome profiling. We will assess their associations with the following clinical outcomes: psychopathological symptoms, cognitive impairment, and level of social functioning.

The use of the author’s VR-MST tailored to SZ patients will help to evaluate the psychological and biological changes associated with this extrapharmacological therapeutic intervention in the treatment of SZ. Training in this form, combining an intervention based on MST tailored in terms of structure (constant directing and restoring attention to the task, along with the elimination of elements potentially increasing the expression of psychotic symptoms, i.e. long periods of silence) carried out in an engagement-enhancing and attention-supporting VR environment (minimizing the influence of the uncontrolled environment on the course of the intervention), has not yet been described in the literature in relation to this group of patients. VR-MST is a novel adaptation and combination of the well-known therapeutic techniques of mindfulness-based stress reduction (MBSR) and VR. This intervention is based on a synthesis of current knowledge and clinical experience with MBSR programs and VR use, including our own experience in combining VR and mindfulness techniques [32, 33]. The session begins by anchoring the experience in the body on a breathing exercise and then proceeds to direct nonjudgmental attention to visual and auditory stimuli. The innovation of our method consists of adapting mindfulness training to the level of functioning of SZ patients by directing attention to the external world, involving visual and auditory perception. Because of the susceptibility of patients with SZ to the risk of triggering symptoms when experiencing silence, we felt that it was necessary to continuously guide subjects’ attention by introducing relatively short intervals in the narrative. The above adaptations were based on clinical experience, the author’s own pilot study [33] and evidence from research on mindfulness groups for SZ patients [24]. The main function of VR in our study is to provide an appropriate, fully controlled, reproducible visual and auditory environment as a basis for training; additionally, the use of VR increases the ability of individuals with attention deficits (typical for SZ) to focus on training content by reducing distractions present in the natural environment. Along with mindfulness instruction and group sessions, this addresses a clear need for structure and relation in therapeutic interventions designed for individuals with SZ [37].

**The aim of the project** is to assess and compare the impact of an authors’ own, innovative VR-MST and a classic non-VR-MST on the following dimensions of clinical outcome as well as the levels of stress-related markers and gene expression in a group of SZ patients.

#### Our general hypotheses are as follows

1) Both groups undergoing mindfulness training will demonstrate greater improvements in the dimensions of clinical outcomes, stress-related markers and stress-induced transcriptional alterations than the TAU group.

2) The VR-MST group will show greater improvements in the dimensions of clinical outcomes, stress-related markers and stress-related gene expression as compared with the non-VR MST group.

**Fig. 2:**
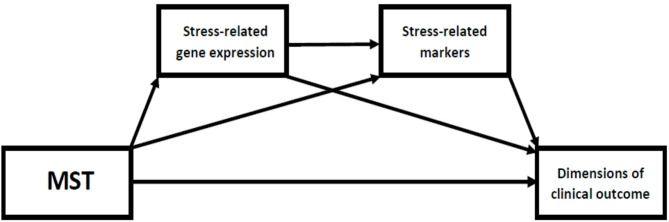
Associations we aim to evaluate in our study. MST– mindfulness skills training

**Our specific hypothesis** is that improvement in terms of dimensions of clinical outcome associated with the mindfulness intervention will be mediated by changes in stress-related markers and gene expression (Fig. 2).

### Research concept and design

To answer the research questions posed, an experimental study with a linear mixed model 3 × 2 design will be conducted: three types of interventions (VR-MST vs. non-VR-MST vs. TAU) as a between-group factor and two measurements (pre-intervention vs. post-intervention) as a repeated-measures factor. The study will measure several psychological-psychiatric and biological dependent variables to determine the status of participants before and after the therapeutic procedure.

Participants diagnosed with SZ according to the ICD-10 and DSM-5 diagnostic criteria will be recruited. Patients will be included if their psychotic symptom severity will not exceed 75 points on the PANSS (moderately ill) [38] and they will be between the ages of 25 and 50 years. With the use of block randomization, the subjects will be randomly assigned to either the VR-MST group, the non-VR MST group or the control group (TAU). Inclusion criteria comprise: (1) SZ diagnosis according to ICD-10 and DSM-5 criteria, (2) psychotic symptom severity below 75 points on the PANSS (moderately ill) (Leucht et al., 2005), (3) Age of the patients between 25 and 50 years old. Exclusion criteria comprise: (1) psychiatric diagnosis other than SZ, (2) acute inflammatory condition or infection present within the last month prior to study inclusion, (3) using non-steroidal antiinflammatory drugs or glucocorticosteroids, (4) diagnosis of severe neurological, autoimmune, inflammatory, or somatic disease, (5) positive or negative symptoms severity that would make the completion of MST instructions impossible, (6) uncorrected visual impairment that prevents the use of VR goggles.

The study will be conducted in cycles, and 12 patients will participate in each cycle. In view of the possibility of drop-out from the study during the procedure, two additional rounds of tests have been planned to supplement the assumed number of groups. Each of the subjects will undergo (fasting) blood collection to determine the level of selected biomarkers. Next, the subjects will fill in questionnaires assessing demographic data, mindfulness skills; and levels of stress, anxiety and depression. In the next research session, cognitive tests will be performed. Additionally, the subjects, apart from collecting the abovementioned anamnestic data, will participate in a psychiatric examination to assess the severity of psychopathological symptoms. Each training session will be simultaneously attended by 4 individuals. In total, participants will attend 24 training sessions (3 × 8 weeks) and 4 group sessions (30 min), where the current training experience and techniques practiced in VR-MST and non-VR-MST will be discussed. Before and after each training session, the participants will fill in short scales to assess their emotional state. Throughout the training period, VR-MST, non-VR MST and TAU subjects will participate in the comprehensive therapeutic program of one of the day wards, and the only difference in therapeutic interventions will be participation in VR-MST or non-VR-MST. After 8 weeks, every person from the VT-MST, non-VR-MST and TAU groups will be examined again with the same set of tests and tools as those used at the initial measurement.

Limitations of our study includes: (1) relatively small sample size (40 patients in each group); (2) relatively high risk of patients drop-out (3) presence of potential confounds (like smoking, BMI, diet, or antipsychotic medication class) that significantly influence cytokine levels and gene expression in schizophrenia (4) generalization of the results limited to the moderately ill patients that can comply with the MST instructions, (5) high number of psychiatric scales included, the burden of assessment might be excessive for such population and may lead to noncompliance.

### Preliminary study results

In one of our previous studies, we utilized VR in the form of 360-degree videos [32]. Its main purpose was to determine whether 360 videos were safe for patients suffering from psychosis and to assess how psychotic patients react to virtual reality situations involving people. Sixteen patients with schizophrenia and twenty-three healthy subjects were recruited for the study. The subjects watched two neutral videos and two social videos via VR goggles. Compared with the control group, patients experienced greater momentary anxiety during social movies, although the severity of social paranoia, as measured by the Social State Paranoia Scale (SPSS), was not significantly different between the groups. The level of emotional arousal between the first neutral and first social movie, operationalized as heart rate, increased in the schizophrenia group but decreased in the control group. In the patient group, a positive correlation was detected between the occurrence of delusional persecutory beliefs on a daily basis, as measured by the Peters Delusion Inventory (PDI), and social paranoia status in VR, as measured by SPSS. These results demonstrate that virtual reality in the form of 360-degree videos affects schizophrenia-diagnosed subjects in a similar way to computer-generated reality, warranting its use in VR-MST.

A pilot study was conducted by our team via an abbreviated version of the procedure described herein [33]. Twenty-five SZ patients participated in the study. Each participant was screened via clinical tools and then again after one month to assess the stability of the measurements. After retesting, the patients participated in a 4-week VR-MST intervention consisting of 12 training sessions followed by the third assessment. All three assessments gauged cognitive functioning with the Addenbrooke’s Cognitive Functioning Scale III, severity of depressive symptoms with the Beck Depression Inventory and the Quick Inventory of Depressive Symptomatology Self Report (QIDS-SR-16) scales, severity of anxiety symptoms with the Beck Anxiety Inventory, and stress intensity with the Perceived Stress Scale severity of psychotic symptoms with the Positive and Negative Symptoms Scale (PANSS-6). Additionally, the subjects completed 7-item affect scales before and after each training session. Patients responded favorably to the intervention. No severe adverse effects were observed during or after the training. Importantly, the participants achieved a significant reduction in symptom severity. This was reflected by the subscales for negative and positive symptoms of the PANSS-6, as well as its total score. There was also a significant increase in cognitive function (ACE III scores). There was a significant increase in the ACE-III score of the examined participants after the completion of mindfulness training compared with the level obtained before training (TAU + VR-MST: *p* = 0.031), whereas the score did not change significantly when only the standard intervention was used (TAU: *p* = 0.308).The analysis of the intensity of the 7 selected emotions, which was based on averaged data from seven training sessions, revealed that compared with the state before training, it significantly changed immediately after training. The patients indicated that sadness, anxiety and anger were less intense, whereas relaxation, joy and surprise increased. The results indicate the safety and acceptability of the intervention among patients with SZ. There were no significant differences in the PANSS results between the initial measurement of patients in the study and the first measurement during the use of TAU, whereas after the VR-MST, the subjects achieved a statistically significant reduction in the severity of symptoms in the overall PANSS score (T2 vs. T3: p = < 0.001) as well as a reduction in negative symptoms (T2 vs. T3: *p* = 0.003) and positive symptoms (T2 vs. T3: *p* = 0.001). The results for depression and anxiety symptoms tended to decrease between the first and second measurements, as well as between the second and third measurements (except for the PSS-10).

Building on the promising findings of prior studies, the authors employed an alternative idiographic approach, the Reliable Change Index (RCI), which facilitates analysis from an individual-change perspective. The effectiveness of Mindfulness Skills Training in Virtual Reality (MST-VR) was evaluated in an article recently accepted for publication in *Psychiatria Polska* [39]. The results indicated that 20 patients (80%) demonstrated improvements across a range of domains, including general symptom severity, positive and negative symptoms, stress, anxiety, depression, and cognitive functioning. Notably, individual cases revealed deterioration in anxiety (2 patients, 8%) and stress (1 patient, 4%). Compared with standard monographic statistical methods, the RCI demonstrated enhanced sensitivity in detecting individual changes. These findings suggest that MST-VR is a safe and beneficial adjunctive treatment for individuals with schizophrenia.

### Research methodology

#### VR-MST and non-VR-MST procedures

The VR‒MST procedure will be conducted via OCULUS QUEST 2 wireless VR goggles in groups of 4, three times a week for 8 consecutive weeks. The subjects will be seated in swivel chairs that create a comfortable environment and opportunities for 360-degree viewing without changing their body position. Prior to each training session, an emotion and mood survey will be conducted via the VAS. Once the subject is seated and wearing goggles, the researcher will turn on a 360-degree video with a resolution of no less than 6 K, depicting a relaxing natural landscape environment with accompanying sounds, followed by an audio track with instructions containing elements of mindfulness training synchronized with the depicted image.

High-quality recording of natural environment will be prepared by a company specializing in recording 360° VR videos in natural landscapes. Moreover, it was agreed that there would be no people in the recording, and the environment would be dominated by greenery and would contain dynamic elements (movement of trees, clouds, and water). The instructions based on attentional skills training procedures will focus on practicing nonjudgmental visual and auditory observations, with visual landscape and soundtrack elements selected by the subject. The general instructions will encourage each individual to make a choice without specific suggestions. In addition, the manual will include messages about conscious directing of attention and guidance on observational activities, as well as the potential for momentary distractions– of a normalizing nature for such events. Breathing relaxation will be performed at the beginning and end of each session. Both procedures should take up to 30 min.The non-VR-MST procedure was prepared to resemble the VR-MST intervention as much as possible. Nonjudgmental attention will be trained not using VR, but in natural settings, however the sitting position and the same audio recording will be used. The aim of this strategy is to determine the effect of VR-MST in relation to non-VR-MST.

Each time after the training, the subject will assess his or her current emotional state on a VAS scale. The first training sessions will be preceded by information on how the training will work and an explanation of what the subjects’ task is. In addition, once every two weeks, a group discussion of the training will be conducted, with particular attention to the motivations for undertaking the proposed exercises while participating in the VR and non-VR sessions.

This research will include a collection of biological markers and psychological and psychiatric measurements. The assessments (except for a short evaluation of emotional state) will take place at two time points with an interval of approximately 9 weeks (pre-post measurement).

#### Psychological and psychiatric assessment

The following scales will be used for the psychological and psychiatric measurements of the variables:

1. Positive and Negative Syndrome Scale (PANSS) and Brief Negative Symptom Scale (BNSS) for the assessment of positive and negative symptoms. The PANSS is a rating scale that evaluates a multidimensional array of positive and negative symptoms in patients with schizophrenia. It consists of positive, negative and general psychopathology scales. The patient is rated on 30 different symptoms from 1 to 7. The severity of presented symptoms is assessed by a psychiatrist and takes approximately 45 min [38, 40].

The BNSS is organized into 5 subscales (anhedonia, asociality, avolition, blunted affect, and alogia). The BNSS is an instrument designed for negative symptom assessment by Kirkpatrick [41].

2. The Montgomery-Åsberg Depression Rating Scale (MADRS) and the Quick Inventory of Depressive Symptomatology-Self-Report (QIDS-SR) will be used to stratify the severity of depressive symptoms. The MADRS consists of 10 items evaluating core symptoms of depression. The symptom rating is based on a clinical interview. The total score ranges from 0 to 60. The QIDS-SR is a 16-item self-report scale used to measure depressive symptom severity. It was derived from the 30-item Inventory of Depressive Symptomatology (IDS) [42]. The internal consistency was high for the QIDS-SR (Cronbach’s alpha = 0.85–0.87).
3. The Perceived Stress Scale 10 (PSS10) will be used to assess the severity of stress. The PSS 10-item version is a self-report instrument rated on a scale ranging from 0 to 4. The total score is the sum of all the points (0–40) [43].
4. The Simulator Sickness Questionnaire (SSQ) to assess simulator sickness symptoms. The SSQ questionnaire created by Kennedy and colleagues, in the Polish adaptation by Biernacki and colleagues, is a self-evaluation measure [44]. It enables subjective assessment of simulator sickness prevalence. Symptoms such as nausea, disorientation, and oculomotor disturbances are scored on a 4-point scale (never, slightly, moderate, or significant). The instrument consists of 26 questions. In the first part of the SSQ, current medical status (physical activity, alcohol consumption, sleep duration) and last use of VR are assessed.
5. The Clinical Global Impression - Severity scale (CGI-S) and Improvement scale (CGI-I) will be used to assess the severity of symptoms and response to treatment. The CGI is a clinician-assessed global summary measure of the patient’s functioning. The CGI comprises two companion one-item measures evaluating the following: (a) severity of psychopathology from 1 to 7 and (b) change from the initiation of treatment on a similar seven-point scale [45].
6. MATRICS Consensus Cognitive Battery (MCCB), Five-Point Test, Trail Making Test (TMT B), Verbal Fluency Test (VFT). Neuropsychological tests to assess cognitive function. The MCCB comprises ten cognitive tests that measure seven discrete cognitive domains: processing speed, attention/vigilance, working memory (both verbal and nonverbal), verbal learning, visual learning, reasoning and problem solving, and social cognition in schizophrenia patients. In validation studies, the MCCB has shown excellent reliability and significant correlations with measures of functional capacity [46]. The TMT provides clinicians with measures of a variety of mental abilities, including letter and number recognition, mental flexibility, visual scanning, and motor function. The TMT is composed of 2 parts, A and B [47]. The VFT is a simple test for evaluating lexicon retrieval and executive function. During the task, participants are given 1 min to produce as many unique words as possible within a given category. The number of unique correct words in each task is the participant’s score. Two tasks are typically examined: semantic fluency (category fluency) and letter fluency (phonemic fluency). In the first task, the aim is to retrieve words in the given semantic category: a wide one (e.g., animals) or narrow one (e.g., sharp objects). In the second task, participants are asked to list words starting with a given letter, e.g., F, A and S. The five-point test is a simple, nonverbal, figural fluency test. The subject is presented with sheets with a grid of squares, each containing 5 dots. The task consists of creating as many unique patterns as possible by connecting 2 to 5 dots in a specified time (usually 2–3 min). The actual test is preceded by instructions for the subject and the presentation of two example designs. This test serves as a nonverbal assessment of executive functioning [48].
7. The Cognitive-Attentional Syndrome Questionnaire (CAS-1) will be used to assess cognitive attentional syndrome. The CAS-1 questionnaire consists of 16 items. The first two, assessed on a scale from 0 to 8, are questions concerning the frequency of rumination and worry as well as concentration on threats. A further six items, assessed on a scale from 0 to 8, concern maladaptive behaviors used to cope with negative emotions and/or thoughts, e.g., thought and situation avoidance, drinking or substance abuse, and attempts to control thoughts or emotions. The last eight items, scored on a scale from 0 to 100, relate to positive and negative metacognitive beliefs that are at the core of the cognitive attentional syndrome, e.g., “worrying too much could harm me” or “worrying helps me cope”. The total score can range from 0 to 128, with a higher score indicating a greater degree of cognitive-attentional syndrome. The Polish version was prepared in cooperation with the author of the original scale and translated into Polish via the back-translation procedure [49].
8. The Hamilton Anxiety Rating Scale (HAM-A) will be used to measure anxiety symptoms. The HAM-A consists of 14 items, each defined by a set of symptoms, and measures both psychological anxiety (i.e., mental agitation and psychological distress) and somatic anxiety (i.e., physical complaints related to anxiety). Each item is scored on a scale from 0 to 4, with a total score ranging from 0 to 56.
9. Short Form of the Five Facets Mindfulness Questionnaire (FFMQ-SF) to assess mindfulness competency. The FFMQ-SF consists of 24 items and has a Polish adaptation with fine psychometric properties [50]. The five facets of mindfulness are observing, describing, acting with awareness, refraining from judging the inner experience and nonreactivity to inner experience. The original FFMQ was created by Baer [51], and its shortened version was created by Bohlmeijer [52]. There is a strong positive correlation between the FFMQ-SF-S results and the coping mechanisms of self-regulation and well-being as well as a strong negative correlation with rumination, neuroticism and depression.
10. Visual Analogue Scale (VAS) to measure the intensity of selected emotions. The VAS is a short version of a rating scale that reports currently experienced emotional states on a scale ranging from 0 to 10. The scale provides an assessment of seven specific internal states: sadness, joy, anger, relaxation, agitation, confusion, and fear.
11. The Functional Assessment Short Test (FAST), the Sheehan Disability Scale (SDS), and the Social and Occupational Functioning Assessment Scale (SOFAS) will be used to assess the level of functioning. The FAST is an instrument for assessing psychosocial functioning. It consists of 24 items (scores 0–3) that assess impairment or disability in six specific areas of functioning: autonomy, occupational functioning, cognitive functioning, financial problems, interpersonal relationships and leisure. The total score is a sum of all the points. Higher results represent worse functioning. The SDS is a composite of three self-rated items designed to measure the extent to which three major domains are functionally impaired by psychiatric or medical symptoms. The SDS assesses functional impairment in three major life domains: work, social life/leisure activities, and family life/home responsibilities. SOFAS– a scale measuring psychosocial functioning, clinician-administered, based on a clinical interview with the patient. The score ranges from 0 to 100 points. A rating of overall psychological functioning between 91 and 100 indicates the highest level of functioning in a wide range of activities. The SOFAS does not consider the overall severity of a patient’s psychological symptoms.
12. The World Health Organization–Five Well-Being Index (WHO-5) will be used to assess well-being. The WHO-5 is a short self-reported measure of current mental wellbeing. The WHO-5 has been found to have adequate validity in screening for depression and in measuring outcomes in clinical trials [53].

#### The biological parameters assessed in the study will include the following

1. the level of brain-derived neurotrophic factor (BDNF) and its fractions (pro, mature),
2. levels of inflammatory markers, e.g., CRP, hs-CRP, TNF-alpha, TNFα, sTNFR60, sTNFR80, IL-6, and IL-8
3. levels of the oxidative stress markers (TBARS)
4. cortisol level
5. transcriptome analysis (RNA-seq) via next-generation sequencing (NGS)

The abovementioned biological markers were selected on the basis of their significant value in the neurobiology of schizophrenia [7, 8, 15, 54–56] and their association with the mindfulness response [57–59]. There are several rationales for the relatively high number of inflammatory markers evaluated in our project for the following reasons: (1) the abovementioned proteins play a particularly important role in the pathophysiology of schizophrenia [6–8, 15, 55]; (2) in addition to their involvement in inflammatory processes, these substances are involved in psychological stress response mediation [60], and (3) inflammatory markers play crucial roles in the stress response model [3] and in the mindfulness model of biological mechanisms according to Black [22]. In addition, if our transcriptome analysis reveals a significant role of new markers, their concentration will be assessed in the serum. To date, our team has conducted numerous research projects evaluating inflammatory markers and oxidative stress indicators in psychiatric patient populations: [61–66].The team responsible for conducting the transcriptome analysis proved the reliability of the above methodology by confirming the capacity to perform RNAseq on relatively small sample size groups in the following publications [67, 68].

RNA sequencing: Ribosomal RNA will be removed with Globin-Zero Gold Removal Kit (Illumina). The mRNA will be enriched with polyA RNA (mRNA) using oligo(dT) beads. After enrichment, the mRNA will be fragmented randomly by adding a fragmentation buffer, then the cDNA will be synthesized on mRNA template utilizing random hexamers primers and reverse transcriptase. The custom second strand synthesis buffer (Illumina), dNTPs, RNase H and DNA polymerase I will be added to initiate the second-strand synthesis. After the series of terminal repair, adaptors ligation, the double-stranded cDNA library will be completed through the size selection and PCR enrichment. The polyA transcriptome libraries will be sequenced on NovaSeq6000 (Illumina) sequencer with the following parameters: PE 150 (paired ends) and 40 M clean reads which give a > 12 Gb of raw data per each sample.

Brain-derived neurotrophic factor levels and levels of inflammatory markers, oxidative stress indicators and stress hormones will be performed by immunoenzymatic techniques based onready-to-use ELISA or colorimetric kits when appropriate (according to the manufacturer’s protocols).

We acknowledge that several potential confounding factors—such as body mass index (BMI), diet, smoking habits, and the type of antipsychotic medication—may significantly influence cytokine levels and gene expression profiles in individuals with schizophrenia. Elevated BMI has been shown to correlate with increased peripheral inflammation, potentially impacting immune-related gene expression [69]. Dietary patterns also play a key role, as nutritional intake can modulate immune function and inflammatory responses, thereby affecting cytokine signaling pathways [70]. Furthermore, the class and type of antipsychotic medication may exert immunomodulatory effects, independently influencing cytokine profiles and gene regulation [71]. Lastly, smoking, which is more prevalent among individuals with schizophrenia, has been associated with alterations in cytokine levels and neuroinflammatory markers [72]. In our research project, certain confounding variables such as smoking and body mass index (BMI) will be statistically controlled due to their potential impact on the outcomes. While not all confounders were directly accounted for in the study design, we acknowledge their relevance and recommend that future studies incorporate these factors both in the planning phase and in statistical analyses.

### Data analysis

Measurements will be compared both between the groups and in a repeated measures analysis scheme (pre-post). If the nature of the data and the assumptions made allow it, a mixed model analysis of variance will be developed, combining both of the above comparisons. Depending on needs and possibilities, correlation or regression methods (multiple regression, logistic, mediation or moderation analysis) and advanced structural equation models or network analysis will also be used. The direct hypothesis will be tested via path analysis according to the methodology of Robinson [73]. Machine learning methods (a penalized logistic regression model combined with nonparametric bootstrapping) will be used to produce classifiers for identifying biomarkers and clinical variables associated with mindfulness therapy outcomes. We will analyze this dataset via a penalized regression method that considers multicollinearity between variables and performs variable selection while computing model performance at the same time.

The proposed subgroup sizes, with the assumed correlation between repeated measurements of 0.5 and a sought-after effect size f = 0.25 (based on the lowest effect sizes for measured parameters in the meta-analyses of Khoury [17] and Hodan-Caudevilla [19]) will achieve a test power of 0.99, which will also be maintained if up to 25% of participants in each subgroup would drop out of the study. To handle missing data, we will use the last-observation-carried-forward (LOCF) method. Level of statistical significance at 0.05 will be considered acceptable. R software will be used for data processing (R Core Team (2025)).

## Conclusions

Contemporary psychiatric research on severe mental disorders prioritizes biological and pharmacological interventions, reflecting a longstanding focus on these approaches. In contrast, investigations into nonpharmacological treatments, especially for serious mental illnesses, remain relatively rare. Although the importance of improving pharmacotherapy is unquestionable, there is a risk of underestimating the role of psychotherapy and psychosocial interventions in facilitating the social reintegration of individuals with severe mental illness. Such interventions may directly enhance quality of life and improve treatment outcomes. In this study, our aim is to explore and promote evidence-based approaches in the design of nonpharmacological interventions for patients suffering from schizophrenia.

The aim of the project is to assess and compare the effects of innovative, authors’ own VR-mindfulness skills training (VR-MST) and classic MST (non-VR-MST) on the dimensions of clinical outcome (psychopathological symptoms, cognitive impairment and level of social functioning) as well as levels of stress-related biomarkers in a group of schizophrenia patients. This project offers an innovative approach to understanding the impact of a novel VR-MST technique on schizophrenia patients’ psychopathology, well-being and functioning, with a special emphasis on its role in reducing stress and its biological markers.

The authors offer the results of scientific seminars held by a multicenter and multidisciplinary team in the form of a research project ready to be implemented, adhering to the principles of evidence-based medicine. These findings could subsequently warrant the recommendation of nonpharmacological interventions as valid treatments for individuals with schizophrenia.

Previous studies have demonstrated that mindfulness-based interventions can reduce stress, improve affect regulation, and have beneficial effects on cognitive performance in schizophrenia patients [74, 75]. Moreover, the incorporation of virtual reality into therapeutic settings has been shown to increase participant engagement and enhance the immersive quality of interventions, potentially leading to stronger therapeutic effects [76, 77].For example, research in related domains has reported improved outcomes in emotional regulation and stress reduction when VR is used to augment traditional therapeutic approaches [78, 79].

By combining mindfulness with immersive VR, we expect our intervention to surpass the effects observed in conventional mindfulness-based interventions. In addition, our focus on both clinical and biological outcomes provides a more integrative framework for evaluating treatment efficacy [80].

The potential practical implications of this research are significant. If effective, VR-MST could become a scalable and accessible tool for community-based or outpatient psychiatric settings, helping to reduce the reliance on pharmacological treatment alone [81]. Furthermore, such an intervention may appeal to younger patients or those with limited motivation for traditional therapies, due to its interactive and engaging format [1]. The digital nature of VR-based interventions may also allow for remote delivery and self-paced training modules, which are especially relevant in the context of modern healthcare systems striving for flexibility and personalization [82].

Our expected outcomes align with existing research showing that mindfulness-based interventions, particularly when combined with technology-enhanced delivery methods, can positively impact cognitive and emotional functioning in individuals with schizophrenia. Previous studies have demonstrated that digital and interactive methodologies may boost therapeutic engagement and improve accessibility for individuals with severe mental illness. For instance, the review by Mancone [1] underscores the efficacy of digital health tools in enhancing health-related outcomes through user-centered and immersive experiences. These findings validate the methodological direction of our study and emphasize the need for further investigation into how such tools can be adapted to meet the needs of clinical populations.

Looking ahead, future research should include randomized controlled trials comparing VR-MST with other nonpharmacological and pharmacological approaches. Longitudinal studies would also be necessary to examine the durability of treatment effects and their real-world applicability in terms of social reintegration and occupational functioning. Additionally, exploring potential moderators such as baseline symptom severity, cognitive capacity, or previous experience with mindfulness could help tailor interventions more effectively [83, 84].

## Data Availability

No datasets were generated or analysed during the current study.
